# The Effect of Dietary *Saccharomyces cerevisiae* on Growth Performance, Oxidative Status, and Immune Response of Sea Bream (*Sparus aurata*)

**DOI:** 10.3390/life12071013

**Published:** 2022-07-08

**Authors:** Ahmed F. Fath El-Bab, Sultan A. M. Saghir, Ibrahim Atta Abu El-Naser, Salwa M. M. Abo El-Kheir, Marwa F. Abdel-Kader, Reem S. Alruhaimi, Haifa A. Alqhtani, Ayman M. Mahmoud, Mohammed A. E. Naiel, Ali Ali El-Raghi

**Affiliations:** 1 Department of Animal Production, Faculty of Agriculture, Damietta University, Damietta 34517, Egypt; ahfarouk74@gmail.com (A.F.F.E.-B.); atta19812000@yahoo.com (I.A.A.E.-N.); Salwaaboelkheir6@gmail.com (S.M.M.A.E.-K.); ali21384@yahoo.com (A.A.E.-R.); 2 Department of Medical Analysis, Princess Aisha Bint Al-Hussein College of Nursing and Medical Sciences, Al-Hussein Bin Talal University, Ma’an 71111, Jordan; sultan_a1976@yahoo.com; 3 Central Laboratory for Aquaculture Research, Sakha Aquaculture Research Unit, Department of Fish Diseases and Management, A.R.C., Giza 12411, Egypt; marwa.abdelkader@vet.kfs.edu.eg; 4 Department of Biology, College of Science, Princess Nourah bint Abdulrahman University, Riyadh 11671, Saudi Arabia; hsalrhemi@pnu.edu.sa (R.S.A.); haalqhtani@pnu.edu.sa (H.A.A.); 5 Physiology Division, Zoology Department, Faculty of Science, Beni-Suef University, Beni-Suef 62514, Egypt; ayman.mahmoud@science.bsu.edu.eg or; 6 Department of Life Sciences, Faculty of Science and Engineering, Manchester Metropolitan University, Manchester M1 5GD, UK; 7 Department of Animal Production, Faculty of Agriculture, Zagazig University, Zagazig 44519, Egypt

**Keywords:** *Sparus aurata*, *Saccharomyces cerevisiae*, *Vibrio parahaemolyticus*, growth, immunity

## Abstract

The objective of this study was to evaluate the beneficial effect of *Saccharomyces cerevisiae* (SC) on growth, intestinal morphometric characteristics, blood indices, redox balance, expression of immune-related genes, and their involvement in disease resistance in sea bream (*Sparus aurata*). Three hundred healthy sea bream fingerlings were allocated into equal four groups (15 fish per hapa). The first group was served as a control and received a basal diet, while the other three groups were fed diets containing 1, 2, and 4 g/kg diet SC, respectively. At the end of week 16, the daily weight gain, specific growth rate, and feed utilization were significantly higher in the SC2 and SC4 groups than the control (*p* < 0.05). SC dose-dependently improved intestinal morphology, and the 4 g/kg diet significantly increased dry matter, crude fat, and crude protein percentage of body composition when compared with the control group. The 4 g/kg SC boosted innate immune response and phagocytic activity, and all SC-supplemented diets improved total protein, glucose, triglycerides, and urea concentrations, as well as intestinal digestive enzymatic activities. All estimated oxidative markers were significantly enhanced in the group that received 4 g/kg SC when compared with the control and other SC groups (*p* < 0.05). Feeding the fish a diet supplemented with 4 g/kg SC markedly regulated the expression of *HSP70*, *IGF1*, and *IL-1β* genes. In addition, the 4 g/kg SC-supplemented diet was the most effective in protecting the fish against *Vibrio parahaemolyticus* challenge. In conclusion, SC-enriched diet improved growth performance, intestinal morphology, redox homeostasis, and immune response of *S. aurata* with the 4 g/kg concentration as the most effective.

## 1. Introduction

Several challenges are now confronting marine aquaculture. Microbial infection outbreaks and excessive feeding costs are the two primary constraints that the farmed fish sector may suffer [1]. Moreover, farmed fish are often subjected to different types of stressors as a result of farm management and environmental variables, which may result in oxidative stress [2,3] and immunosuppression [4,5]. Therefore, the use of a proper feeding system that supports fish immunity might have a substantial beneficial influence on boosting performance and promoting the profitability of cultured fish throughout the whole production cycle [6]. There is a clear link between improving gut microbiota biodiversity and fish productivity and health [7]. The manipulation of gut microbiota throughout the use of dietary supplements enriched with beneficial microbes (probiotics) is currently receiving a significant scientific and commercial interest. The beneficial microorganisms colonize the host gut and multiply with several beneficial impacts on both the host and its ambient environment [8]. In aquaculture, the probiotics examined for use were found to include both Gram-positive and Gram-negative bacteria as well as bacteriophages, unicellular algae, and yeast [9,10].

*Saccharomyces cerevisiae* (SC) is one of the most popular probiotics in extract or whole cell form. Yeast has the capability to stimulate bile and regulate acidic pH, as well as being free of all types of plasmid-encoded antibiotic resistance genes and non-pathogenic [11,12]. Dietary SC is the most common single cell protein that is incorporated into aqua feeds with the aim of efficiently reducing the need of protein of animal origin [13]. It is effective in promoting the growth efficiency and feed utilization in seabass fry [14], rainbow trout frys [15], Nile tilapia [16,17], and gilthead seabream [18]. It can contribute to improving gut health by producing some energy substrates for intestinal cells from the yeast cell. Moreover, there are many benefits related to brewer’s yeast nutritional nucleotides, such as rapid repair of intestinal mucosal surfaces with relative elongation as well as the improvement of intestinal mucosal flora [19]. The cell wall components of SC, including chitin, mannoprotein, and glucan are immunostimulants and their growth-promoting effects have been evidenced in sea bream [20]. Enriching rainbow trout diets by mannan-oligosaccharide has (MOS) improved the average daily gain and feed efficiency as well as the density of posterior gut microvilli [21,22]. *β*-glucan-supplemented diets improved growth performance of *Labeo rohita* [23], but failed to increase the average daily gain in Nile tilapia [24]. However, some studies have observed that MOS had no influence on the growth of gilthead seabream and catfish [25,26].

The present study was conducted to examine the potential effects of SC on growth performance, intestinal morphometric parameter, hemato-biochemical parameters, oxidative status, and immune-related genes and to determine its role in the disease resistance in cultured sea bream (*Sparus aurata*).

## 2. Materials and Methods

### 2.1. Experimental Rearing Conditions and Diet Preparation

The experimental diets were prepared by thoroughly mixing the dry ingredients of each diet, followed by adding 200 mL of water per kg diet. The mixture was blended to make a paste and pelleting was carried out by passing it through a laboratory pellet machine with a 1 mm diameter. The resulting wet pellets were dried at room temperature and were stored in plastic bags at 4 °C until use. All formulated diets were prepared to meet the macronutrient and essential amino acid requirements of sea bream according to the published literature [27]. The components of the formulated diet were listed in Table 1 and the chemical composition of diet samples was evaluated using AOAC techniques [28]. New test pellets were made weekly to maintain the actual SC incorporated in the experimental diets. The survival of the supplemented SC in the diet was assessed following storage at 4 °C by culturing diluted samples on yeast-extract-peptone-dextrose (YPD) agar plates. The medium contained 2% peptone, 1% yeast extract, 2% glucose, and 1.5% agar [29].

Apparently healthy fingerlings of *S. aurata* (average weight 31.23 ± 1.2 g) were obtained from a private farm EL-Mussallas (Damietta, Egypt), transported and were maintained in 1000 L tanks for adaptation for 15 days, and fed the basal diet. At the end of acclimatization period, apparently healthy fish were allocated randomly into 20 enclosures (hapa) (1 × 2 × 1.25 m) 15 fish per hapa. The experimental fish were distributed into four equal groups within 5 replicates as follows: The 1st group was fed the basal diet (control), while the 2nd, 3rd, and 4th groups were fed diets containing 1, 2, and 4 g/kg SC, respectively, for 16 weeks. Fish were hand-fed to apparent satiation twice per day (09:00 and 15:00 h), and feed intake was recorded daily. Water quality parameters were monitored twice per week during the trial, and a photoperiod regime (12:12 h light:dark), water temperature (19 °C), pH (7.18), salinity (27.45 ppt), NO_2_ (0.029 mg/L), NO_3_ (0.037 mg/L), NH_3_^+^ (0.038 mg/L), DO_2_ (5.7 mg/L), K^+^ (2.47), Na^+^ (215.11), Mg^2+^ (59.85), Ca^2+^ (73.15), SO_4_ (168.1), Cl^−^ (175.01), HCO_3_^−^ (4.38), and CO_3_^2−^ (0.00). All physicochemical characteristics evaluated in water were considered appropriate for *S. aurata* growth as reported by Faggio et al. [30].

### 2.2. Growth Performance and Feed Utilization Indices

Hand feeding was used to ensure that no food remained on the aquarium bottom. Daily remaining unconsumed food was carefully removed from each aquarium 30 min after each feeding period. The actual feed intake (FI) was calculated by subtracting the dry mass of leftover diet from the total afforded feed. The body weight of *S. aurata* was recorded at the beginning and end of the experiment. Growth performance was determined and feed utilization was calculated as follows:

Weight gain (WG) = final body weight (g) − initial body weight (g)

Feed conversion ratio (FCR) = feed intake (g)/weight gain (g)

Specific growth rate (SGR) = 100 × [ln final body weight (g) − ln initial body weight (g)/duration of feeding (day)]

### 2.3. Blood Sampling

Blood samples were collected from the caudal vertebral vein of 15 fish per group, anaesthetized with 40 mg/L olive oil according to Feldman et al. [31]. Each collected blood sample was divided into two tubes, one containing EDTA for hematological examination, and the other was used for serum separation by centrifugation at 4000 rpm for 15 min. The collected serum was stored at −20 °C until used.

### 2.4. Hematological Analysis

The erythrocytes and leukocytes were counted according to the method described by Stoskopf [32] using hemocytometer and Natt-Herrik solution. Hemoglobin concentration was determined using the cyanomethemoglobin method. In this method, hemoglobin was converted into methemoglobin using ferricyanide and cyanide ion. Methemoglobin is a stable red compound and can be measured colormetrically. The micro hematocrit method was used for estimation of PCV% [33]. Differential leukocytic count (DLC) was determined using a thin blood film that was air-dried, fixed with methanol for 3–5 min, and stained with Giemsa for 8–10 min. The stained film was rinsed with distilled water and left to dry. The white blood cells were counted and absolute DLC was calculated according to Thrall et al. [34] formula:

Absolute DLC = number of each white cell × number of total leukocytic count/100

### 2.5. Determination of Phagocytic Activity

The bacterial strain (1 × 10^6^ CFUs/mL *Aeromonas hydrophila* A5216) was supplied by Department of Fish Diseases and Management, Sakha Aquaculture Research Unit, Central Lab. for Aquaculture Research (Kafr El Sheikh, Egypt). The bacterial strain was selected based on the study by Reyes-Becerril et al. [35]. Leukocyte phagocytic function was determined following the method of Cai et al. [36] after slight modifications. Briefly, 0.5 mL of blood was mixed with 0.25 mL *Aeromonas hydrophila* suspension in glass tubes, which was maintained at 28 °C in a water bath for 30 min. The tubes were shaken every 10 min and then centrifuged [36]. Blood smears were carried out in duplicates and stained with Giemsa/May-Grunwald [37]. The number of leukocytes that engulfed bacteria was counted and represented as the percentage of total leukocytes in the smear.

Phagocytic activity (PA) = number of phagocytic cells with engulfed bacteria/total number of phagocytic cells × 100.

Phagocytic index (PI) = total number of engulfed bacteria in 100 phagocytic cells/100.

### 2.6. Biochemical Assays

Serum total protein (TP) was determined with commercially available kit (Spectrum, Cairo, Egypt). Albumin (Alb) was assayed with a reagent kit supplied by Biodiagnostic (Giza, Egypt), and globulin was calculated mathematically. Activities of aspartate aminotransferase (AST) and alanine aminotransferase (ALT), and concentrations of creatinine, urea, triglycerides, and cholesterol were assayed with Biodiagnostic (Giza, Egypt) kits. Glucose level was determined with a kit supplied by Vitro Scient (Cairo, Egypt). Cortisol was measured by fluorescence immunoassay rapid quantitative test using a commercial kit and FIA meter (Finecare FIA meter plus, Wondfo Biotech Co., Guangzhou, China). Lysozyme activity was assayed using ELISA kit (Sigma, St. Louis, MO, USA) according to the manufacturer’s instructions.

### 2.7. Determination of Malondialdehyde (MDA) and Antioxidants

Superoxide dismutase (SOD), catalase, glutathione peroxidase (GPx), and MDA were assayed in serum according to the methods described by Nishikimi et al. [38], Aebi [39], Paglia and Valentine [40], and Ohkawa et al. [41], respectively, using reagent kits supplied by Biodiagnostic (Egypt).

### 2.8. Determination of Digestive Enzymes Activity

After the fish were anesthetized with eugenol (1: 10,000; Shanghai Reagent Corp., Shanghai, China), the gut was directly dissected from 5 fish per hapa (25 fish per treated group) and stored in liquid nitrogen. The samples were homogenized using an electric homogenizer (XHF-D, Xinzhi, China) and the homogenate was centrifuged under cooling for 15 min at 5000 rpm. The supernatant was collected for assaying lipase and amylase activities according to the methods of Moss and Henderson [42] and Caraway [43] using kits supplied by Spectrum (Cairo, Egypt) and Biodiagnostic (Giza, Egypt), respectively.

### 2.9. Gene Expression

The fish were dissected and liver samples (10 per group) were collected in sterile Eppendorf tubes and maintained in liquid nitrogen for RNA isolation. One hundred milligrams were used for total RNA extraction using Trizol (iNtRON Biotechnology, Seongnam-Si, Korea) according to the manufacturer’s instructions. The Nanodrop (UV-Vis spectrophotometer Q5000, Quawell, San Jose, CA, USA) was used to confirm the quality and quantity of the isolated RNA. Samples with OD260/OD280 ≥ 1.8 were used for cDNA synthesis using a SensiFAST™ cDNA synthesis kit (Bioline, Nottingham, UK), following the manufacturer’s instructions. Insulin-like growth factor I (*IGF-I*), interleukin-1β (*IL-1β*), and heat shock protein 70 (*HSP70*) specific primers were used to amplify the selected genes (Table 2) with *β-actin* as a housekeeping gene. Amplification was carried out using TOP real™ preMIX SYBR Green qPCR master mix (Enzynomics, Daejeon, Korea) on Stratagene MX300P PCR system. The data were analyzed according to the 2^−∆∆CT^ method [44].

### 2.10. Morphometry of Intestinal Villi

The histological examination was adopted according to Gewaily et al. [45]. Five fish were randomly selected from each treatment. After deep anaesthesia, the intestine samples were obtained and cut into pieces of approximately 0.5 cm. The samples were fixed in 10% neutral buffered formaldehyde for 24 h, dehydrated in ascending grades of alcohol, cleared with xylene, and embedded in paraffin wax. Five-μm thick sections were cut using a rotatory microtome (RM 20352035; Leica Microsystems, Wetzlar, Germany) and stained with hematoxylin and eosin (H&E), then examined using a BX50/BXFLA microscope (Olympus, Tokyo, Japan).

### 2.11. Experimental Infection

A previously identified virulent strain of *Vibrio parahaemolyticus* was used for the experimental infection. Total bacterial count, determined using the drop plate method, was used in demonstration of the inoculum dose for the experimental studies according to Cruickshank et al. [46]. The LD_50_ was assessed before the final challenge up to 10 days [47]. At the end of the 16th week of the feeding trial, fish of each group were maintained in 12 glass aquaria (60 L) (*n* = 30 per diet) for acclimatization for 7 days. *V. parahaemolyticus* was propagated in brain heart infusion broth (BHI) at a temperature of 25 °C for 24 h. The bacterial suspension was performed by the addition of a sterile saline solution [48]. Each fish was intraperitoneally injected with 0.2 mL of LD_50_ dose of *V. parahaemolyticus* strain, which was determined previously (2 × 10^7^ CFUs). All fish groups fed the control and experimental diets were challenged in triplicates. All injected fish were observed for a period of 7 days post-inoculation. Mortalities were recorded daily and freshly dead fishes were moved for further post-mortem examination.

### 2.12. Statistical Model and Analysis Procedure

Prior to statistical analysis, any heterogeneity of variances was adjusted using the appropriate data transformation when necessary. For estimated significance variance between means, one-way analysis of variance (ANOVA) was used, followed by Tukey’s post-hoc test. The statistical significance was accepted at a probability value less than 0.05. Kaplan-Meier survival data were analyzed using the log-rank (Mantel-Cox) test with GraphPad Prism 8 (GraphPad Software, San Diego, CA, USA).

## 3. Results

### 3.1. Effect of Dietary SC on Growth Performance

FBW, DWG, and SGR in the SC groups were significantly higher than the control group (*p* < 0.05). FCR and PER in SC2 and SC4 groups were significantly lower than other treated groups and the control (*p* < 0.05). The changes in FBW, DWG, SGR, and PER were non-significant in SC2 when compared with SC4 (Table 3).

### 3.2. Effect of Dietary SC on Whole Body and Muscle Composition

The dry matter, crude fat, and ash contents in the SC groups were significantly higher than those in the control (*p* < 0.05), while the dietary treatments decreased the moisture content significantly (*p* < 0.05) when compared with the control. The crude protein in the SC4 group was higher than the control group (*p* < 0.05). Non-significant differences were detected between the control group and both of the SC1 and SC2 groups (Table 4).

### 3.3. Effect of Dietary SC on Intestinal Morphology

**Figure 1:**
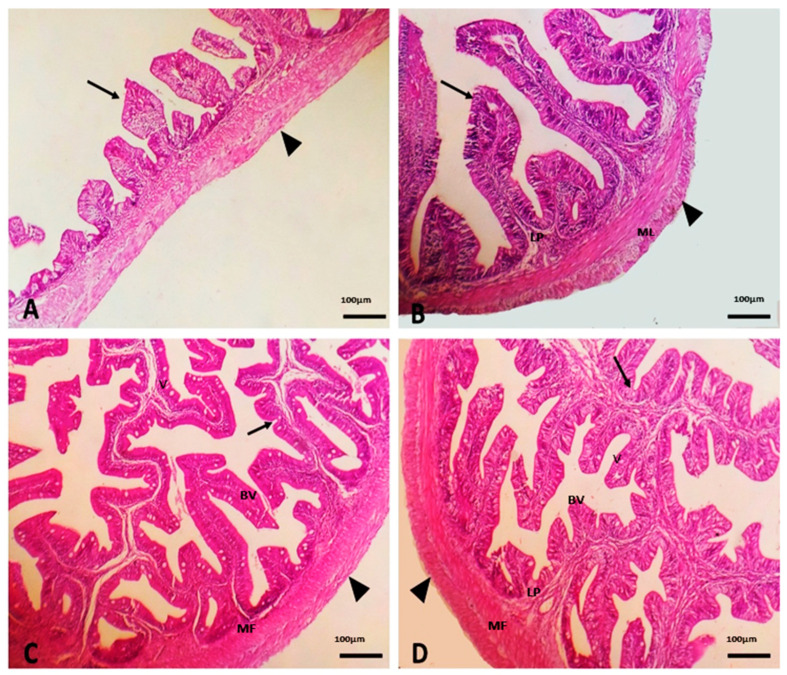
Photomicrograph of the intestine of sea bream showing the histological structure in the control (**A**) and 1 (**B**), 2 (**C**), and 4 g/kg (**D**) SC-supplemented groups. The intestine appeared intact and was formed of intestinal wall (arrowhead) and protruding intestinal villi (arrow). The SC-supplemented groups showed gradual increase in the length and branching of villi. SC supplemented diets reduced vacuole count and increased the thickness of muscular layer. MF, mucosal folds; LP, lamina propria; ML, muscular layer; BV, branchial villus; and V, vacoule.

The histological structure of the sea bream intestine in the control group appeared intact and consists of intestinal wall and protruding intestinal villi. The wall of the intestine comprised of mucosa, submucosa, muscularis, and serosa. The intestinal villi appeared intact and were lined by simple columnar epithelium around the connective tissue core (Figure 1A). The intestinal villi appeared normal, non-branched, and slightly increased in length in the SC1-supplemented group. The best morphological appearance was found in the SC4 group followed by the SC2 (Figure 1B–D). The SC2-supplemented group had slightly branching and longer villus length as well as a thicker epithelial cell wall. Furthermore, the SC2 group had a greater vacuole count. The SC4-supplemented group had the longest villus length with more branches, and the thickest epithelial cell layer with the fewest vacuole count (Figure 1B–D).

### 3.4. Effect of Dietary SC on Hematological and Immunological Variables

The RBC count and MCHC did not alter significantly as a result of the dietary supplementation of SC. HGB and PCV values were significantly higher in the SC2 and SC4 groups than the control (*p* < 0.05). The values of MCV and MCH were significantly higher in the SC4 group than the SC1 group and the control (*p* < 0.05), whereas non-significant differences were shown between the SC1 and SC2 groups (*p* < 0.05). WBCs were significantly higher in the SC4 group than the SC1, SC2, and control groups (*p* < 0.05). Monocytes, basophils, and eosinophils were not significantly affected by the dietary treatment. However, lymphocytes were significantly higher in the SC4 groups than the control group and other SC groups. Lysozyme and phagocyte activities as well as IgM levels were significantly higher in the SC2 and SC4 groups than the control (*p* < 0.05; Table 5).

### 3.5. Effect of Dietary SC on Circulating Biochemical Parameters

Transaminases (ALT and AST) and cortisol were not significantly affected by dietary SC (*p* < 0.05). TP, Alb, and globulin were significantly higher in the SC2 and SC4 groups than the control group. Glucose, TG, Chol, and urea concentrations were significantly higher in the SC2 and SC4 groups than the control group (*p* < 0.05), whereas non-significant differences were observed between the SC2 and SC4 groups (*p* < 0.05). Amylase activity was higher in the SC4 group than the control and other treated groups. Lipase activity was higher in the SC2 and SC4 groups than the control group (*p* < 0.05), while non-significant differences were observed between the SC1 and SC2 groups (*p* < 0.05; Table 6).

### 3.6. Effect of Dietary SC on Redox Status

SOD and CAT activities were significantly higher in the SC4 groups than the control and other SC groups (*p* < 0.05), whereas non-significant differences were observed between the SC1 and SC2 groups. Conversely, MDA levels were remarkedly lower in the SC2 and SC4 groups than the control and SC1 groups (*p* < 0.001; Table 7).

### 3.7. Effect of Dietary SC on HSP70, IGF1, and IL-1β Expression

**Figure 2:**
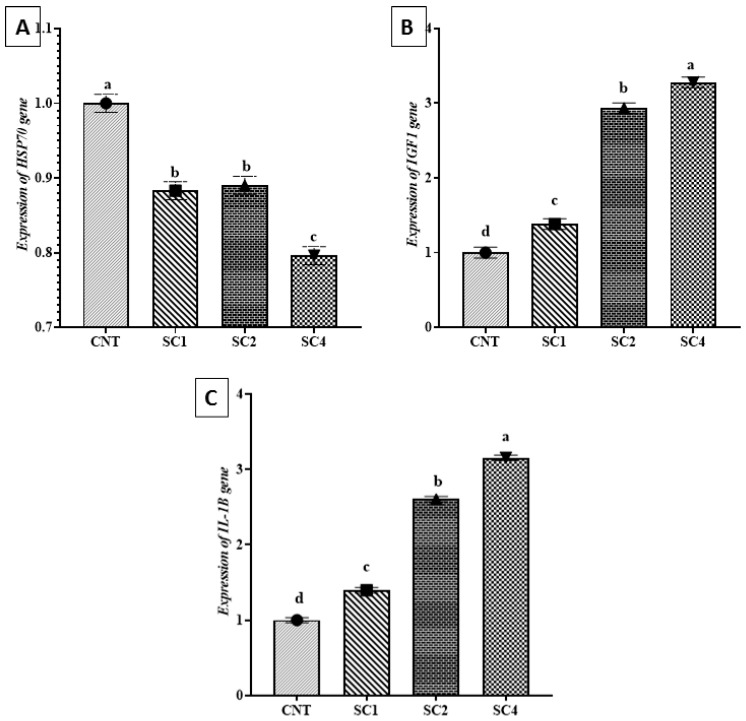
Effect of SC supplementation on the expression levels of (**A**) *HSP70*, (**B**) *IGF1*, and (**C***) IL-1β* in sea bream. *HSP70*, heat shock protein; *IGF1*, insulin-like growth factor 1; *IL-1β*, interleukin-1β. Data are mean ± SEM; ^a,b,c,d^ values with different superscripts in the same column differ significantly (*p* < 0.05).

*HSP70* was downregulated and *IGF1* and *IL-1β* genes were upregulated significantly in the treated groups when compared with the control as depicted in Figure 2A–C. In comparison with other treated groups, the SC4-supplemented fish displayed significant upregulation of both *IGF1* and *IL-1β* and downregulation of *HSP70* expression (Figure 2).

### 3.8. SC Increased Survival Rate in Sea Bream Challenged with V. parahaemolyticus

**Figure 3:**
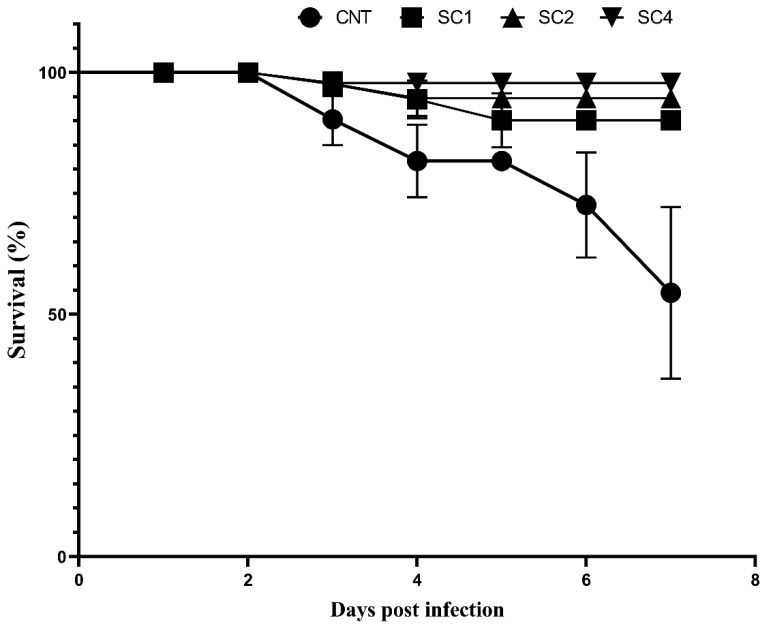
SC increased survival rate of sea bream challenged with *V. parahaemolyticus*. Data are mean ± SEM, N = 3.

*S. aurata* survival rate was calculated for 7 days after *V. parahaemolyticus* challenge. The number of deaths started at day 3 post infection and SC supplementation increased the survival rate. The survival rate of the SC4 group was significantly higher than the control, SC1, and SC2 groups (*p* < 0.05). Non-significant differences were observed between the control group and both the SC1 and SC2 groups (Figure 3).

## 4. Discussion

In aquaculture, one of the most prominent methods to improve the growth performance and enhance the health status is the use of probiotics as nutritional supplements, which is both protective and eco-friendly [49]. Merrifield et al. [50] reported that probiotics could be beneficial to the host by boosting the immune response and intestinal microflora. SC is considered an important source of products with probiotic activity [51]. SC is rich in valuable nutrients, which can play roles in promoting growth performance, such as vitamin B, proteins, amino acids, and carbohydrates [52,53]. In particular, β-glucan and MOS found in the yeast cell wall have the capability of improving growth performance and animal health [54]. In the present study, 2 and 4 g/kg SC-supplemented diets showed a growth-promoting effect on sea bream evidenced by the improved FBW, DWG, SGR, and feed utilization (FCR and PER). In accordance, Zhang et al. [55] reported that 1–3% yeast-enriched diets improved DWG and SGR in shrimps. The addition of 1% yeast products in *L. vannamei* diets enhanced growth performance and FCR [56]. The study by Chaitanawisuti et al. [57] showed that the dietary supplementation of yeast nucleotides at levels of 1% and 2% improved the growth performance and feed utilization of *Babylonia areolata*. The growth performance could be linked to the improved feed utilization and digestibility of nutrients. Probiotics could participate in digestive processes by producing enzymes, such as amylases, lipases, and proteases [58,59]. In the present study, SC (4 g/kg) promoted the secretion of digestive enzymes, which might improve nutrient digestibility, leading to improvement of feed efficiency and growth performance in sea bream. In contrast, under cage culture system, diets enriched in yeast extract did not improve growth performance or FCR in hybrid tilapia [60]. In addition, Jarmołowicz et al. [59] demonstrated that supplementing diets with yeast extract did not significantly affect the growth rate of juvenile European pikeperch. The discrepancies among these studies could be attributed to the difference in culture systems, fish species, physiological conditions, and nutrient composition of the diets. Growth is recognized as a polygenic and environmentally controlled trait with the most influential genes being those of IGF1 and growth hormone [61]. IGF1 secretion is affected by insulin, growth hormone, and other hormones as well as metabolic and nutritional conditions [62]. As a result, IGF1 gene expression could be strongly regulated by nutrients. In the current study, supplementation of the sea bream diets with SC, particularly the 4 g/kg diet, resulted in a significant increase in IGF1 mRNA, which supported the aforementioned results related to FBW, SGR, and FCR.

Morphometric parameters related to intestinal villi dimensions can help in predicting the digestion and absorption mechanisms in the fish gut [63]. SC-supplemented diets showed a gradual increase in the length and branching of villi. The beneficial effect of SC on intestinal morphometric parameters in the current study could be attributed to MOS found in the cell wall of yeast, which showed a positive effect on the morphology of villi and microvilli in several fish species [64]. Furthermore, dietary live yeast has the potential to produce polyamines, which help in enhancing fish gut health [65]. This effect might be caused during the growth phase in the SC-treated groups since the integrity and density of the intestinal mucosa and intestinal villi require a long time to develop. In the same context, Islam et al. [66] reported that the intestinal mucosal fold, lamina propria width, enterocyte count, and the number of goblet cells were improved in Nile tilapia fed diet supplemented with 4 g/kg SC. Moreover, previous studies have demonstrated that yeast improved the intestinal morphological parameters in the Nile tilapia [67] and carp [68].

Imbalanced feed composition can undoubtedly have a negative impact on the function, viability, and body composition of the fish. Enzyme production could be increased by dietary yeast supplementation, which promotes the nutrient composition and growth of aquatic animals. In this study, positive effects of the SC-supplemented diets on protein, dry matter, crude protein, moisture, ash, and lipids content in the whole body of sea bream have been observed. Similarly, Abu-Elala et al. [12] showed positive effects of yeast supplementation on the nutrient composition and growth of Nile tilapia. Moreover, Ayiku et al. [52] reported similar results in shrimp, and Ebrahimi et al. [69] reported increased crude protein content in common carp fingerlings that received a diet supplemented with a combination of MOS and *β*-glucan (2.5 g/kg). These findings indicated that the physiological performance of aquatic animals could be enhanced by the yeast culture supplementation.

Hematological and biochemical markers can provide useful information on the health status of aquatic organisms [70]. The improved hematological (HGB, PCV, MCV, and MCH) values in the current study indicated that SC feeding can protect fish from anemia. Significant improvements in the WBC and LYM following SC supplementation highlighted the immune-stimulatory effects of yeast and positive effects on the health of sea bream. Globulin is a crucial component of the innate immune system of aquatic animals to protect the body against invasive organisms [71]. Here, dietary SC ameliorated not only globulin, but also serum TP and Alb. In accordance, dietary SC may serve as an immuno-stimulant for sea bream. Zhang et al. [72] showed a significant improvement in blood protein of grass carp fed diets supplemented with yeast culture, which modulated immune activities. Dawood et al. [73] reported increased TP in *Nile tilapia* fed probiotic-based diets, suggesting immuno-modulatory effects of SC.

Yeast possesses anti-obesity effect and is therefore a healthy food additive [74]. In obese adults, Jung et al. [75] reported that yeast supplementation can reduce and prevent the accumulation of abdominal fat. In the current study, the groups fed SC showed a significant decrease in serum Chol and TG, demonstrating the beneficial effect of supplementing the diet with SC on lipid profile. In this context, Ayiku et al. [52] reported a significant decrease in serum Chol and TG in shrimp fed diets containing 2% yeast. In aquatic animals, increased serum cortisol or glucose is an indicator of stress [76]. In addition to serum lipids, SC ameliorated glucose levels but had no effect on cortisol. These findings pointed to the ability of SC to improve energy homeostasis and protect against stress in sea bream.

Reactive oxygen species (ROS) are produced from metabolic activities in animals and when increased to overcome the antioxidant capacity of the cells, oxidative stress occur. The activity of antioxidant enzymes is used to examine the antioxidant status in aquatic animals in response to stressful conditions as well as oxidative stress resulting from surplus ROS generation [77]. SOD converts superoxide to hydrogen peroxide and oxygen and thus represents the first line of defense [78], and CAT catalyzes the decomposition of hydrogen peroxide [79]. In addition to its damaging effect on cellular proteins and DNA, excessive ROS can provoke lipid peroxidation, leading to membrane destruction and cell death [80]. In the present study, SOD and CAT activities were significantly increased and MDA was decreased in the SC-treated groups, demonstrating improved redox homeostasis. Similar to our findings, Yang et al. [81] reported significant improvement in CAT and SOD activities in shrimps fed diets supplemented with the yeast *Rhodosporidium paludamentum*. Yuan et al. [68] showed enhanced CAT and SOD activities in serum of grass carp fed yeast hydrolysates-supplemented diets. The yeast cells contain vitamins, which may beneficially affect antioxidant defenses and immune system of aquatic animals [55]. Along with the improved antioxidants, SC improved serum AST and ALT activities, enzymes involved in amino acid catabolism, and their high activities in serum reflect liver damage. In this study, it was demonstrated that SC had no adverse effect on sea bream.

Lysozyme activity is considered a biomarker for examining the bactericidal activity of the nutritional value of diets [82]. Saurabh and Sahoo [82] suggested that increased lysozyme activity in aquatic animals may be linked to abundance of immune system-related cells, which constitute the main source of proteolytic enzymes. In the present study, there was a significant increase in lysozyme activity in the SC-fed groups, which could be a result of increased release from the lysosomes or the proliferating phagocytes. Phagocytosis, an immune defensive process against infectious diseases [83], was significantly improved in SC-fed groups. Additionally, IgM which plays multiple roles in maintaining lymphoid tissue architecture and B cell survival [84] was significantly increased in the 4 g/kg SC-fed group. These findings supported the vital role of SC in activating the immune responses in sea bream and improving its resistance to pathogens. Fish fed with 4 g/kg SC-supplemented diet in this study exhibited significant upregulation of the pro-inflammatory cytokine *IL-1β*, which has a vital role in adjusting the immune response of fish [85] similar to what have been reported by Yang et al. [86] in the orange-spotted grouper. Furthermore, the 4 g/kg SC diet-fed fish showed significant downregulation of *HSP70*, which is expressed in fish exposed to environmental stressors [87], pinpointing the anti-stress activity of SC.

The farming of sea bream can potentially increase the risk of infectious disease outbreaks owing to the number of individuals living in close proximity to each other. Therefore, a proactive approach to health management must be adopted at each farm location to mitigate and minimize these problems. *Vibrio* species in aquaculture under intensive culture causes serious economic loss by increasing mortality [88]. Feeding sea bream with diets and then challenging it with pathogenic bacteria has been demonstrated as a useful method to examine disease resistance. SC supplementation enabled sea bream to resist bacterial infection and increased survival rate. Similar to the present results, Ayiku et al. [52] showed significant resistance in shrimp fed 2% yeast against *V. harveyi* infection. Increased resistance of sea bream against *V. parahaemolyticus* infection provided sufficient evidence for the dose-dependent role of SC in stimulating the immune response against pathogenic infections. The study by Yang et al. [86], showing that supplementation grouper diets with SC extract improved *V. harveyi* challenge, added a further support to the observed immunomodulatory role.

## 5. Conclusions

This study introduced information on the beneficial effect of SC-supplemented diet in improving growth performance, energy homeostasis, and immune response in sea bream. Supplementing sea bream diets with 4 g/kg SC could be an effective intervention that can positively affect growth performance, intestinal function, hematological indices, redox homeostasis, and immune response of sea bream in cultures. Therefore, SC could be used as a potential probiotic in sea bream farming.

## Figures and Tables

**Table 1:** Diet formulation and chemical composition of the experimental diets.

Ingredient	%
Fish meal (Crude protein, 70%)	58
Wheat flour	27.4
Fish oil	11.2
Lecithin	1
^a^ Minerals + Vitamin mix	0.4
Fish protein soluble	2
Total	100
**Chemical composition**	
Crude protein	45.1
Crude Lipids	18.3
Ash	8.8
Starch	20.3
^b^ Gross Energy, GE, kJ/g	22.7

^a^ Providing, per kg of mix: Vitamin E, 5.8 g; vitamin K3, 3.3 g; thiamin, 3.3 g; riboflavin, 6.6 g; pyridoxine (as pyridoxine hydrochloride), 3.3 g; niacin, 16.6 g; folic acid, 3.3 g; vitamin B12 (cyanocobalamin), 0.01 g; d-biotin, 0.1 g; vitamin c (ascorbic acid), 33.3 g, calcium pantothenate, 13.3 g; Cu as copper sulfate, 3 g; I as calcium iodine, 0.4 g; Co as cobalt carbonate, 0.3 g; Mn as manganese sulfate, 10 g; zinc oxide, 30 g; sodium selenite, 0.08 g; calcium, 0.8 g. ^b^ Estimations of GE were based on protein, lipid, and carbohydrate content analyzed in feed raw materials multiplied by the energy content of the previous: GE = protein × 23.62 kJ/g + lipid × 39.52 kJ/g + carbohydrates × 17.2 kJ/g.

**Table 2:** Primers used for qRT-PCR.

Gene	Forward (5→3′)	Reverse (5→3′)	GenBank Accession No.
*β-actin*	CGACGGACAGGTCATCACCA	AGAAGCATTTGCGGTGGACG	AF384096.1
*IGF1*	AGTGCGATGTGCTGTATC	CAGCTCACAGCTTTGGAAG-	EF563837.1
*HSP70*	AATGTTCTGCGCATCATCAA	CCAACCTTTTTGTCCAATCC	EU805481.1
*IL-1β*	GGGCTGAACAACAGCACTCTC	TTAACACTCTCCACCCTCCA	115592467

**Table 3:** Effect of dietary SC on growth performance and feed utilization of sea bream (*S. aurata*).

Parameters	Control	Dietary Treatments			SEM	*p*-Value
		SC1	SC2	SC4		
IBW (g)	31.233	32.600	31.233	31.833	1.254	0.0963
FBW (g)	109.737 ^b^	126.053 ^a^	129.460 ^a^	129.767 ^a^	1.287	<0.001
DWG (g/day)	0.700 ^c^	0.834 ^b^	0.877 ^a^	0.874 ^a^	0.012	<0.001
SGR (g/d^−1^%)	1.120 ^c^	1.207 ^b^	1.270 ^a^	1.254 ^a^	0.013	<0.001
FCR (g/g)	2.624 ^b^	2.525 ^bc^	2.502 ^c^	1.916 ^a^	0.038	<0.001
PER (g/g)	1.312 ^a^	1.179 ^b^	1.125 ^c^	1.136 ^bc^	0.017	<0.001

IBW, initial body weight; FBW, final body weight; DWG, daily weight gain; SGR, specific growth rate; FCR, feed conversion ratio; PER, protein efficiency ratio; ^abc^ values with different superscripts in the same row differ significantly (*p* < 0.05).

**Table 4:** Effect of dietary SC on whole body chemical analysis (on dry weight basis) of sea bream (*S. aurata*).

Dietary Treatments	Dry Matter	Moisture	Crude Protein	Crude Fat	Ash
Control	33.630 ^c^	66.370 ^a^	40.820 ^b^	30.273 ^d^	18.433 ^a^
SC1	35.990 ^b^	64.010 ^b^	46.970 ^ab^	32.916 ^c^	17.870 ^b^
SC2	36.273 ^b^	63.726 ^b^	47.060 ^ab^	35.823 ^b^	17.020 ^c^
SC4	36.993 ^a^	63.006 ^c^	52.260 ^a^	37.396 ^a^	16.970 ^c^
SEM	0.192	0.192	2.991	0.366	0.070
*p*-value	<0.001	<0.001	0.0387	<0.001	<0.001

^abcd^ values with different superscripts in the same row differ significantly (*p* < 0.05).

**Table 5:** Effect of dietary SC on hematological and immunological variables of sea bream (*S. aurata*).

	Control	Dietary Treatments			SEM	*p*-Value
		SC1	SC2	SC4		
Hematological variables						
RBCs (×10^6^ µL)	3.610	3.733	3.583	4.003	0.504	0.4326
HGB (g/dL)	10.986 ^c^	11.636 ^b^	11.950 ^a^	12.200 ^a^	0.080	<0.001
PCV (×10^3^ µL)	35.33 ^c^	36.66 ^bc^	38.33 ^ab^	40.00 ^a^	0.645	0.0046
MCV (fL)	89.07 ^c^	97.22 ^b^	97.71 ^ab^	98.20 ^a^	0.153	0.0077
MCH (pg/dL)	30.44 ^b^	30.45 ^b^	30.79 ^ab^	31.15 ^a^	0.158	0.0397
MCHC (g/dL)	31.00	31.88	31.40	31.26	0.120	0.4421
Immunological variables						
WBCs (×10^6^ µL)	19.273 ^c^	26.140 ^b^	27.717 ^b^	30.707 ^a^	0.867	<0.001
Monocytes (%)	8.00	9.00	9.00	9.33	0.527	0.3700
Lymphocytes (%)	70.33 ^a^	81.00 ^a^	78.00 ^a^	56.00 ^b^	4.847	0.0307
Basophils (%)	0.666	1.000	1.000	0.666	0.235	0.5954
Eosinophils (%)	1.333	1.333	1.000	1.000	0.372	0.8473
Lysozyme (%)	6.850 ^d^	8.960 ^c^	9.303 ^b^	10.303 ^a^	0.087	<0.001
Phagocytic activity (%)	8.160 ^c^	11.136 ^b^	11.206 ^b^	12.036 ^a^	0.098	<0.001
Phagocytic index	1.000 ^b^	1.103 ^ab^	1.213 ^a^	1.243 ^a^	0.044	0.0164
IgM (ng/mL)	3.040 ^c^	4.340 ^b^	4.610 ^b^	5.220 ^a^	0.153	<0.001

RBCs, red blood cells; HGB, hemoglobin; PCV, packed cell volume; MCV, mean corpuscular volume; MCH, mean corpuscular hemoglobin; MCHC, mean corpuscular hemoglobin concentration; WBCs, white blood cells; IgM, immunoglobulin M; ^abcd^ values with different superscripts in the same row differ significantly (*p* < 0.05).

**Table 6:** Effect of dietary SC on biochemical parameters of sea bream (*S. aurata*).

Parameters	Control	Dietary Treatments			SEM	*p*-Value
		SC1	SC2	SC4		
TP (g/dL)	4.906 ^d^	5.020 ^c^	5.206 ^b^	5.300 ^a^	0.015	<0.001
Alb (g/dL)	1.310 ^b^	1.334 ^b^	1.340 ^b^	1.400 ^a^	0.017	0.031
Globulin (g/dL)	3.596 ^d^	3.683 ^c^	3.863 ^b^	3.906 ^a^	0.007	<0.001
Glucose (mg/dL)	12.336 ^d^	14.010 ^c^	15.070 ^a^	15.970 ^a^	0.114	<0.001
TG (mg/dL)	88.020 ^c^	91.256 ^b^	97.560 ^a^	99.733 ^a^	1.607	<0.001
Chol (mg/dL)	91.430 ^c^	97.220 ^b^	104.523 ^a^	107.860 ^a^	1.982	<0.001
Urea (mg/dL)	1.680 ^a^	1.670 ^a^	1.560 ^b^	1.583 ^b^	0.007	<0.001
AST (IU/L)	20.026	20.006	18.763	18.836	0.582	0.310
ALT (IU/L)	30.186	29.213	29.380	28.626	0.359	0.0822
Amylase (U/L)	40.623 ^d^	46.600 ^c^	52.183 ^b^	58.270 ^a^	1.037	<0.001
Lipase (U/L)	34.550 ^c^	49.980 ^b^	51.407 ^ab^	52.883 ^a^	0.758	<0.001
Cortisol (ng/mL)	26.080	25.333	25.626	24.893	0.337	0.0674

TP, total protein; Alb, albumin; TG, triglyceride; Chol, cholesterol; AST, aspartate aminotransferase; ALT, alanine aminotransferase; ^abcd^ values with different superscripts in the same row differ significantly (*p* < 0.05).

**Table 7:** Effect of dietary SC on MDA and antioxidant enzymes in sea bream (*S. aurata*).

Parameters	Control	Dietary Treatments			SEM	*p*-Value
		SC1	SC2	SC4		
MDA (mmol/L)	14.650 ^a^	11.303 ^b^	10.040 ^c^	9.773 ^c^	0.327	<0.001
CAT (U/mL)	11.130 ^b^	11.343 ^b^	11.410 ^b^	12.513 ^a^	0.277	0.0423
SOD (U/mL)	9.470 ^c^	10.100 ^b^	10.290 ^b^	10.716 ^a^	0.105	0.0002

MDA, malondialdehyde; CAT, catalase; SOD, superoxide dismutase; ^abc^ values with different superscripts in the same row differ significantly (*p* < 0.05).

## Data Availability

Not applicable.
